# The British Journals: Surgery

**Published:** 1839-04

**Authors:** 


### SURGERY.

*On the Formation of Moulding Tablets for Fractures, &c.*
By ALFRED SMEE, Dresser at St. Bartholomew's Hospital.

[THE following proposal is ingenious, and seems to offer some advantages over other analogous materials employed in fractures and other surgical diseases.]

The importance of a substance that can be moulded accurately to any part of the body at a moment's notice, must be admitted by every member of the medical profession; yet many difficulties attend the formation of a composition which shall, at the period of its application, be so yielding and soft, that it may take an accurate cast of any part, and when dry, shall still retain the form given to it, and become sufficiently hard to resist external impressions, and at the same time shall be tough, elastic, and devoid of brittleness and much flexibility; and further difficulties present themselves where the capability of its being quickly dried is required. The advantage of lightness and cheapness is also a great desideratum.

As I had frequently noticed that the composition of gum arabic and whiting, when dry, possessed great hardness and toughness, and yet was so free from brittleness that it could scarcely be pounded in a mortar, I was determined to ascertain how far it would answer to make tablets, which might be used to form extemporaneous splints.

For this purpose a piece of coarse sheeting was copiously brushed over on one surface with a thick solution of gum, after which it was covered with a composition made by rubbing whiting with mucilage, continually adding the powder until the whole was of the consistence of a thick paste: a second piece of sheeting was now rubbed over on one side with the solution of gum, and the moistened side applied upon the composition with which the piece of sheeting had been covered, and we thus had two thicknesses of sheeting with an intervening layer of the composition of mucilage and whiting, the thickness of which may be increased or diminished as strength or lightness is desired. The whole was then dried, and formed a tablet about the thickness of slight pasteboard.

This experiment succeeded beyond my most sanguine expectations; for, whilst the tablet remained dry, it was exceedingly hard, and, when sponged over with a little warm water, became so yielding, that, by moulding it with the fingers, a cast could be taken of any part of the body. The hand and knuckles were defined with great accuracy, and I succeeded, by a little management, in taking a cast of the greater part of the face. It is sometimes advisable not to allow the substance to dry upon the part on which it is moulded; but after the depressions and elevations have been traced with the fingers, it should be carefully removed, and partially dried before the fire; and as soon as the texture is sufficiently dry to retain its shape, it may be placed near a stone, or even on the hob of a grate, without fear of corrugating or becoming otherwise deformed. In most cases, however, this drying is quite unnecessary, it being requisite only to envelope the moist tablet with a bandage. A cast thus taken is extremely hard and tenacious, so that when not much thicker than a wafer, it may be struck violently and repeatedly against any hard substance, and not be destroyed. It possessed but slight flexibility, and, after having been bent, it returned to its previous form, showing considerable elasticity. It was neither liable to be torn nor broken; and lastly, it possessed the advantage of lightness combined with durability. Whilst in search of a moulding substance, I thought it advisable to try various compositions, in order that the best might be selected, but none appeared so excellent as that last described.

Of all these preparations, and many others that were tried, few were applicable, and none in all respects equal to the composition of gum and whiting, both of which substances are always easily obtained, and have the additional advantage of cheapness. The solution of gum, which was found most adapted, contained 10 or 12 oz. of gum to the pint of water.

As far as regards the nature and texture of the cloth, it is to be remarked, that linen is stronger than cotton, and less liable to be torn, and therefore to be preferred. Of the various kinds of linen, none moulds so perfectly as moderately coarse old sheeting; for when the tablets were made of finer Irish, they were very inferior in this respect.

The application of these tablets is rather extensive; they may be used with great advantage for all fractures of the metacarpal bones; also for those of the forearm, or even for the humerus. *Lon. Med. Gazette. Feb.* 23, 1839.

---

### *Case of Hernia successfully treated on Dr. O'Beirne's plan.*

By J. G. Wilson, Surgeon to the Bristol General Hospital.

[This case contains no novelty to those who are acquainted with Dr. O'Beirne's valuable papers: we transcribe it for the benefit of those who are not.]

The case occurred in a sailor, æt. 34, subject to a chronic scrotal hernia: strangulation had existed about eighteen hours, when he was visited by Mr. Wilson. "When I saw him, his countenance was anxious; his pulse small and frequent; the tumour was tense, and very tender to the touch; and the skin of the scrotum reddened. He had hiccough, and retched or vomited incessantly; and he had not passed fæces for three days. I could not judge of the nature of what he vomited, as he discharged the contents of the stomach in a bucket of dirty water. I placed him on his back with his knees elevated, and immediately applied the taxis in the usual way, but without avail. I repeated it again and again, using as much force as I thought safe, and as much, indeed, as the painful state of the tumour would admit of, but all in vain. As the symptoms were now urgent, and evidently getting more and more so, I thought of removing him on shore, in order that he might be more conveniently situated for the operation, which I considered it more than probable he would have to undergo; but before doing so, I determined to try the plan of treatment proposed by Dr. O'Beirne, whose very interesting paper, in the Dublin Journal for September last, I had recently read. Accordingly, I procured an elastic tube, such as he recommends, thirteen inches in length; and having straightened it and oiled its extremity, I

introduced it into the rectum. It passed, with little or no obstruction, the whole of its length; but as no flatus escaped, I then attached it to the syringe, and threw up nearly a quart of water, in which two tablespoonfuls of common salt had been dissolved, with an ounce of oil added. This enema quickly returned; but as he was obliged, for want of better convenience, to use the bucket, I was unable to see whether much feculent matter had passed off. On his again lying down I applied the taxis, but no change was perceptible in the tumour. Feeling assured that I could have passed the tube higher had its length allowed me, I sent for the œsophagus tube of the stomach-pump, and introduced it with the same ease, more than three fourths of its length, taking care to pass it very gradually, and upon the least obstruction to attach it to the syringe, when one or two smart strokes of the piston, sending a jet of water through the tube, immediately overcame the difficulty, and allowed it to pass on with ease. When rather more than eighteen inches of the tube had been passed up, some flatus began to escape, and on examining the tumour I found it evidently less tense; the man expressed himself in some degree relieved, and the vomiting ceased. I had withdrawn the tube to allow him to pass off the water which had been injected to facilitate its passage; and this time I observed that nothing but the clear water returned. I then again introduced the tube to the same length, when flatus escaped through it and by the side of it. I then turned the patient on his back, and found the tumour was sensibly diminished, before I began to apply the taxis, and now, with very little difficulty, I was enabled to return the greater portion. About one third of its bulk resisted my pressure for a few minutes longer, and from its feel seemed to be omentum; but in less than ten minutes from the last introduction of the tube, the whole was reduced, and all the urgent symptoms ceased at once.

"The man now requested to be allowed to go to stool, and passed a large quantity of feculent matter." *London Medical Gazette. December*, 15, 1838.

---

### *Astounding Injury of the Brain recovered from.*
By W. ROBERTS, Surgeon, Carnarvon.

[WE copy the following case as we find it in our contemporary, but we utterly disbelieve the facts to be as therein recorded: were they true, the narrative would entail lasting disgrace on all concerned in this horrid proceeding.]

Just seven years to this time I was sent for to attend a young man who had met with an accident in the extensive slate quarries of Mr. Ashton Smith, in this county; while stamping a rock the powder ignited, and the blast went direct to his face; both eyeballs were shattered to pieces, the scalp on the forehead very much lacerated, and above the inner canthus of the left eye was a small hole in the os frontis, fairly through into the brain: upon my arrival I found the person who attends the men at the quarries introducing a grooved director through this hole, and scooping the inside of the skull, bringing out some blackish sludge and a good deal of brain, both cortical and medullary part. *I must confess I had no great hopes of the patient's recovery, and thinking I could do no more mischief than had already been done, being also anxious to know if the brain possessed any sensitive power, I took the director and passed it, in a direct line, until it touched the os occipitis opposite, and then turned it round into different parts; the young man, being quite conscious all this time,* assured me it gave him no pain, and it was only at the hole in drawing out the instrument that he did feel pain. It may be necessary to observe, there was no pressure of the skull on any part of the brain, and no fracture, with the exception of the hole in the os frontis. After dressing the wounds, and giving general directions as to the after treatment, I took my leave, and was not sent for again; the patient got well without any unfavorable symptoms, and it so happened I did not see him from that period until a few months since. The person already alluded to assured me he repeatedly introduced the director afterwards, but it was not at my desire; *from the manner in which the instrument was used at different times, I am satisfied both the hemispheres of the cerebrum must have been broken down, and made a regular puddle of;*

notwithstanding, the young man has been in good health ever since, and not only that, but all the faculties of his mind are quite perfect, as well as his hearing, taste, smell, and feeling; his sight, of course, is totally destroyed. His neighbours have assured me that he is a sensible, shrewd young fellow, a good singer, and remarkable for his memory. I shall content myself by merely stating the facts of this case; what I have here adduced can be attested by living witnesses; the young man himself lives to tell the tale; his name is Griffith Jones; he resides at a small farm called Ty-du, within half a mile of the Old Inn, on the banks of the celebrated Lakes of Llanberris. *Lancet. Jan.* 26, 1839.

---

*On Encysted Dropsy of the Thyroid Gland, with a method of Operation and Cure.* By Congreve Selwyn, m.d., Cheltenham.

[Practical surgeons are well acquainted with this form of bronchocele, although they are too apt to confound it, in treatment, with the common or *cellular* variety, as Dr. Selwyn terms it. In the latter, the specific effect of iodine is well known; in the former, this remedy is generally powerless. The author of this paper appears to have met with many cases of this disease during his practice at Ledbury, and recommends the seton as an infallible remedy.]

I proceed to the operation in this manner: An assistant grasps the tumour on both sides, pushes it forwards, and renders it as tense as possible; I then introduce, at one side of the tumour, a fine and small trocar, about two inches in length by one eighth of an inch in circumference, with a canula to draw off the fluid. In the first instance I used a probe, with a trocar point, to introduce the silk, but I have employed, latterly, a common stocking-needle, armed with one or two threads, and carried it through the canula and out at the opposite side. The period required for the cure is various, according to the bulk of the wen, the state of constitution, disposition to healing, and other obvious circumstances. I have never failed, as yet, by this plan of treatment in a single case. *Lancet. Dec.* 15, 1838.

